# Cronkhite-Canada Syndrome: A Rare Cause of Chronic Diarrhoea in a Young Man

**DOI:** 10.1155/2016/4210397

**Published:** 2016-01-28

**Authors:** Dhrubajyoti Bandyopadhyay, Adrija Hajra, Vijayan Ganesan, Suvrendu Sankar Kar, Debarati Bhar, Manas Layek, Sabyasachi Mukhopadhyay, Cankatika Choudhury, Vivek Choudhary, Prasun Banerjee

**Affiliations:** ^1^Department of Accident and Emergency, Lady Hardinge Medical College, New Delhi, India; ^2^Department of Internal Medicine, IPGMER, Kolkata, India; ^3^Department of Internal Medicine, R.G. Kar Medical College, Kolkata, India; ^4^Lady Hardinge Medical College, New Delhi, India; ^5^R. G. Kar Medical College, Kolkata, India

## Abstract

A young Indian man presented with nine-month history of chronic diarrhea, occasionally mixed with blood and intermittent colicky abdominal pain. He also complained of generalized body swelling for the last three months. On examination, he had diffuse hyperpigmentation of the skin and dystrophic nail changes. Upper and lower gastrointestinal endoscopy revealed multiple sessile polyps in the stomach, small bowel, and colon and rectum. Biopsy of polyps showed adenomatous changes with stromal edema and dilated glands. Cronkhite-Canada syndrome (CCS) was diagnosed and treated with glucocorticoids and enteral nutritional supplementation. There was an associated small intestinal bacterial overgrowth (SIBO) and stool was positive for clostridium difficile toxin. After 12 weeks of treatment, the patient achieved remission. Close correlation with clinical findings, including pertinent ectodermal abnormalities, endoscopic studies, and careful examination of biopsies will ensure a timely and correct diagnosis of CCS.

## 1. Introduction

In 1955, Cronkhite and Canada reported the first example of an acquired nonfamilial syndrome that now bears their names [1]. Patients typically present in the sixth decade of life. It involves tissues arising from the ectodermal germ cell layer but does not appear to be an inherited disorder [2]. It is characterized by the presence of diffuse GI polyposis, dystrophic changes in the fingernails, alopecia, cutaneous hyperpigmentation, diarrhea, weight loss, abdominal pain, and complications of malnutrition [1]. Polyps are found throughout the gastrointestinal tract with characteristic sparing of the esophagus [2]. The etiology of CCS is still obscure, but an autoimmune process has been suggested [3]. Approximately 450 cases of CCS have been reported worldwide [3]. Patients of European or Asian descent are most frequently affected and 75% of reports came from Japan [4].

## 2. Case Report

**Figure 1:**
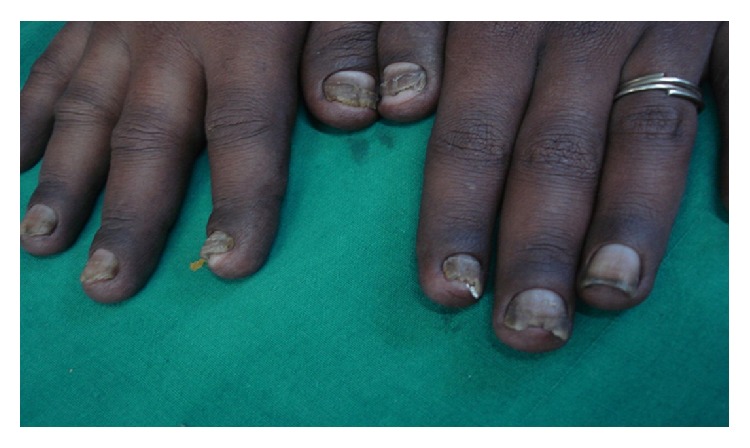
Onychodystrophy of finger nail.

**Figure 2:**
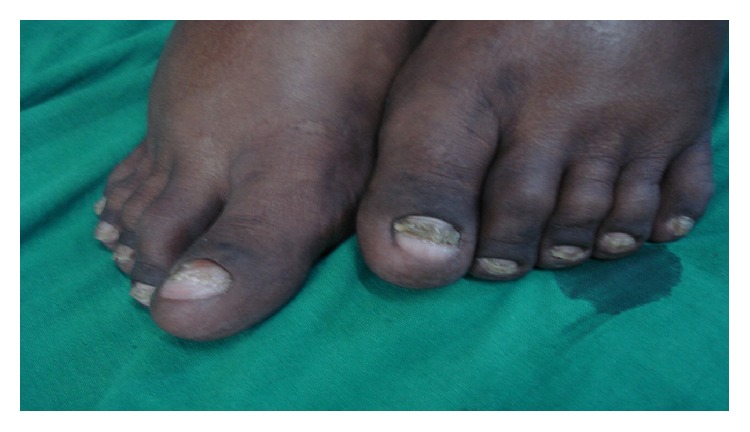
Onychodystrophy of toe nails.

**Figure 3:**
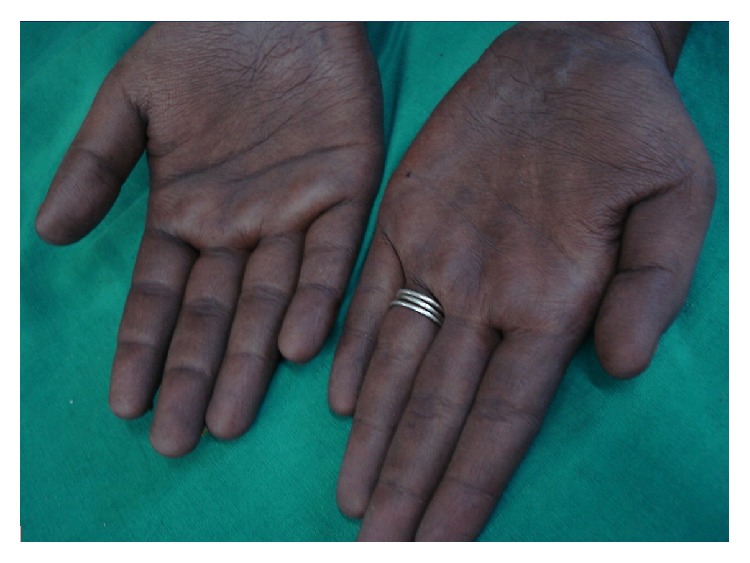
Hyperpigmentation in hands.

**Figure 4:**
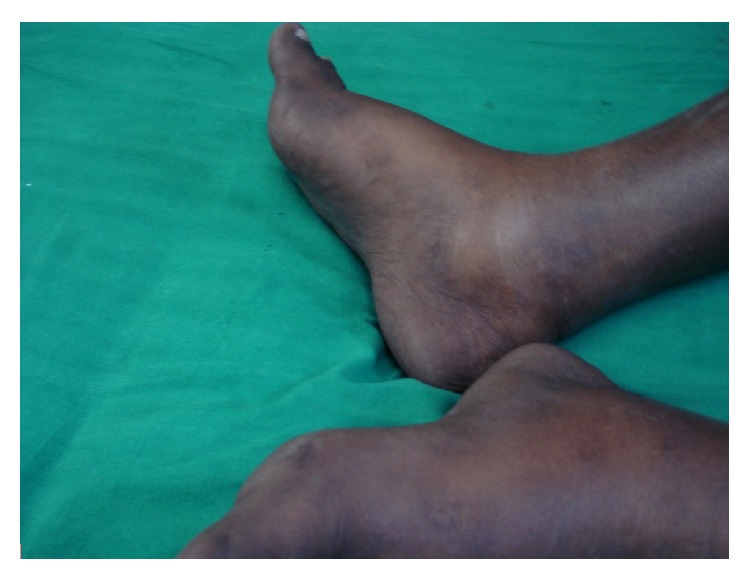
Hyperpigmentation in legs.

A 26-year-old nondiabetic nonhypertensive male patient presented with chronic diarrhea with occasional blood in stool and intermittent colicky diffuse abdominal pain for the last nine months. He also complained of anasarca and dystrophic nail changes for last three months. The patient denied any vomiting and had no significant change in appetite. There was no fever, cough, abdominal lump, or extraintestinal manifestations. On examination, he had mild anemia, dystrophic nail changes of both upper and lower limbs (Figures 1 and 2), and diffuse hyperpigmentation of the skin (Figures 3 and 4). His body mass index (BMI) was 18 kg/m^2^. 

## 3. Investigations

**Figure 5:**
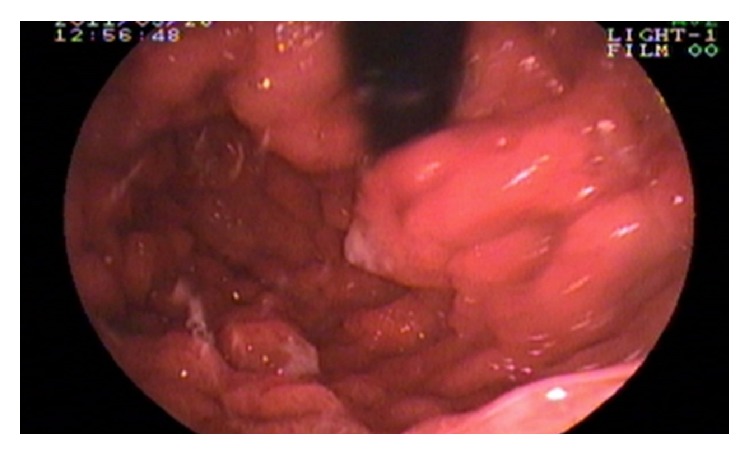
Polyposis in stomach.

**Figure 6:**
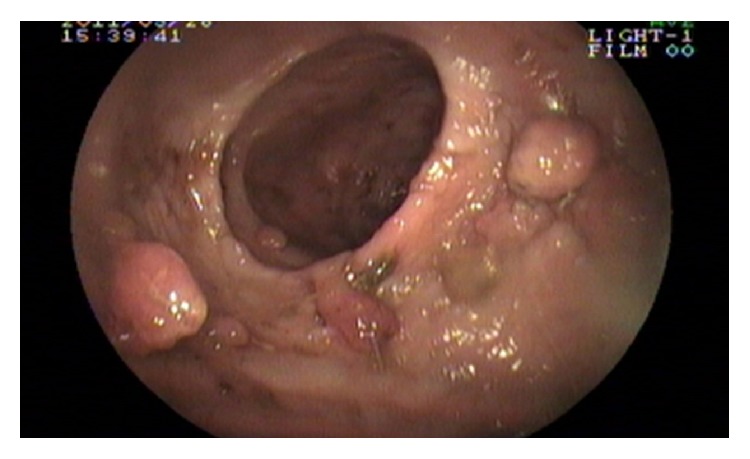
Double lumen enteroscopy showing diffuse polyposis in small intestine.

**Figure 7:**
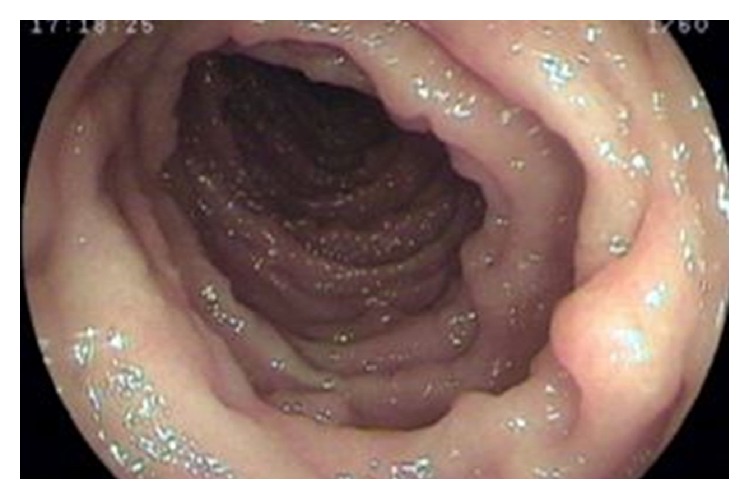
Diffuse polyposis in colon.

**Figure 8:**
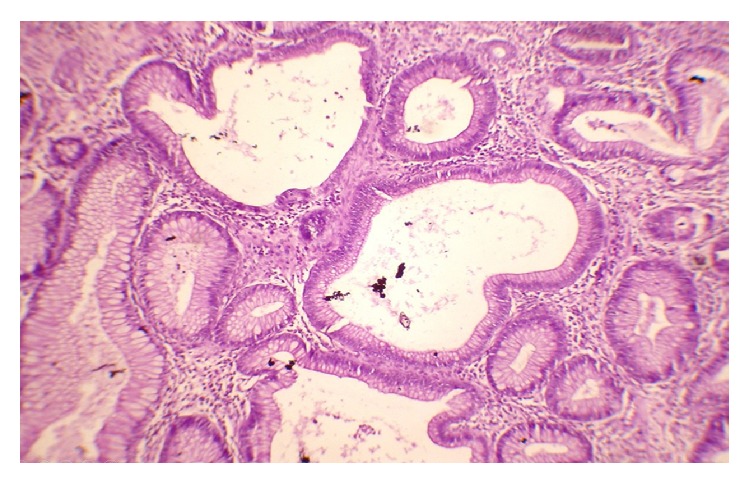
Biopsy showing adenomatous changes with stromal edema and dilated glands.

**Figure 9:**
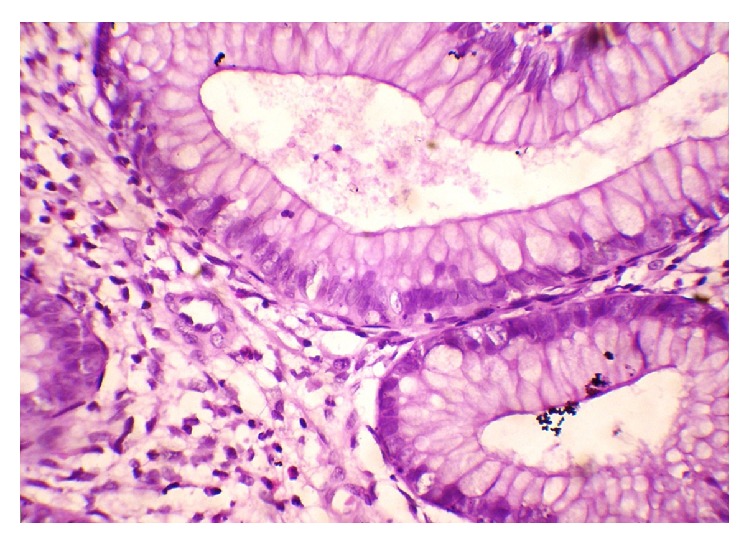
Biopsy showing adenomatous changes with chronic inflammation.

Laboratory examination showed the following: haemoglobin: 9 gm%; total leucocyte count: 6700/cmm; platelet: adequate; serum albumin: 2.7 gm/dL; serum globulin: 2.4 gm/dL; and AST, ALT, and ALP: within normal range. Serum calcium and vitamin D level were low. Serum sodium and potassium, renal function tests, and thyroid function tests were within normal limits. Anti-HIV 1 and HIV 2 antibody, HBsAg, and anti-HCV antibody are nonreactive. Stool for the occult blood test was positive. Ultrasonography of the whole abdomen showed mild thickening of the bowel wall. Chest X-ray and ECHO cardiography were normal. The patient underwent an upper gastrointestinal endoscopy, colonoscopy, and double-balloon enteroscopy to investigate further his condition. The endoscopic evaluation revealed multiple sessile polyps in the stomach, small bowel, and colon and rectum (Figures 5, 6, and 7). Biopsies were taken from few of these lesions. Histopathology of the polyps showed adenomatous changes with stromal edema and dilated hyperplastic glands (Figures 8 and 9). Helicobacter organisms were not seen in gastric or duodenal specimens. Serum antinuclear antibody (ANA) and immunoglobulin gamma 4 (IgG4) were negative. Cronkhite-Canada syndrome (CCS) was diagnosed based on a combination of clinical features and endoscopic and biopsy findings. Breath hydrogen test for SIBO was positive.

## 4. Treatment

The patient was treated with daily oral prednisolone (40 mg/day, tapered over 12 weeks), omeprazole (20 mg twice daily), and antispasmodic for pain abdomen. The stool was positive for clostridium difficile toxin. For SIBO and clostridium difficile infection, he was put on oral metronidazole for 14 days and the patient was improved. He was on enteral nutrition with zinc, vitamin D, and calcium supplementation.

## 5. Follow-Up

The patient improved clinically within 12 weeks. The frequency of diarrhea was reduced, and abdominal pain disappeared. But his nail and skin changes do not improve significantly despite the 12-week treatment. He was discharged with oral medications and advised to attend the monthly outpatient clinic and annual screening colonoscopy to detect any malignant transformation. On the 24th week of follow-up visit, patient gained 2.5 kg weight and stool frequency was on average two times per day. Skin and nail changes still were present.

## 6. Discussions

CCS is a rare and serious disease with rapid progression and high mortality rate. The estimated incidence of CCS is one per million based on the result of the largest study performed to date. The mean age of onset is estimated to be in the sixth decade, with a slight male predominance 3 : 2 in ratio [5]. But the age of our patient was 26 years which is extremely rare in literature.

Diarrhea in CCS is attributable primarily to diffuse small intestinal mucosal injury, but bacterial overgrowth may be contributory. GI polyps are found in 52% to 96% of patients and range in location from the stomach to the rectum [1]. These polyps are hamartomas similar to the juvenile (retention) type, but, unlike juvenile polyposis, the mucosa between polyps is histologically abnormal, with edema, congestion, and inflammation. As is the case with juvenile polyps, there may be foci of adenomatous epithelium that can confer a risk of carcinoma. It is estimated that the risk of colon cancer is approximately 9%, and the risk of adenomas or adenomatous change is 40% [6]. Gastric cancer risk is also increased. Thus, screening of the colon and stomach should be considered. Mortality rate exceeds 50% regardless of therapy [4]. Spontaneous regressions, however, were observed in 5–10% of CCS cases, regardless of treatment [7].

It was originally thought that the epidermal changes were secondary to profound malnutrition as a result of protein-losing enteropathy. Recent findings have called this hypothesis into question; in particular, the hair and nail changes may not improve with improved nutrition as seen in our case after 12 weeks of follow-up [6].

Although this is a nonfamilial disorder of unknown etiology, hypotheses suggest an immune-mediated disorder. Though in our patient IgG4 was negative, tests on some of CCS patients had shown immunostains positive for IgG4 [8].

Optimum therapy for CCS is not known, but several treatment options have been described. Nutritional support, systemic glucocorticoids, azathioprine, anabolic steroids, anti-inflammatory (i.e., mesalamine), histamine-receptor antagonists, cromolyn sodium, and surgical treatment have all been used with varying degrees of success. Unfortunately, controlled therapeutic trials have not been possible because of the rarity of the disease. Individualized treatment depending on the presentation and complications is the best possible option. Also due to the rarity of illness, optimal screening protocols have not been developed. Annual endoscopic surveillance for possible carcinoma screening and investigation for detection of SIBO should be considered to improve mortality and morbidity [3].

The total treatment period is also unknown; recommendations range from 6 to 12 months of combined therapy [9]. The longest surviving patients were alive 15 and 17.5 years after successful surgical treatment [4]. Causes of death are attributable to severe cachexia, anemia, congestive heart failure, embolism, shock, bronchopneumonia, and postoperative complications. One-third of the patients die from intractable nutritional deficiency [4]. However, with improvement in medical therapy and early recognition of the syndrome, the prognosis is now better compared to earlier case reports. But till now it is inconclusive whether the increased survival is due to early detection, leading to lead-time bias or response to therapy.

## 7. Take-Home Messages

One should consider the following.

1. A high index of suspicion and recognition of the characteristic histological findings frequently facilitate a correct diagnosis.
2. Early diagnosis may have prognostically better outcome.
3. Even in younger patient with chronic diarrhea, we should keep in mind this rare diagnosis.
